# A *Staphylococcus* Dominant Nasal Microbiota in Hemodialysis Patients

**DOI:** 10.1016/j.xkme.2025.101079

**Published:** 2025-09-19

**Authors:** Sylvia Wu, Nicholas Tedrow, Abhinav Bhalla, Alex Devito, Sheavonnie Wright, Friederike Selbach, Darshana M. Dadhania, Carol Li, Vesh Srivatana, Jeffrey Silberzweig, John Richard Lee

**Affiliations:** 1 Division of Nephrology and Hypertension, Department of Medicine, Weill Cornell Medicine, New York, NY; 2 Department of Transplantation Medicine, New York Presbyterian Hospital–Weill Cornell Medical Center, New York, NY; 3 The Rogosin Institute, New York, NY

To the Editor:

The anterior nares contain a diverse set of both commensal and pathogenic bacteria. Previous studies have investigated the impact of nasal colonization with a particular focus on *Staphylococcus aureus* in hemodialysis (HD) patients.^1^ Several studies have reported the use of oral rifampin or nasal mupirocin to prevent *S. aureus* carriage and infections.^2^, ^3^, ^4^ However, to the best of our knowledge, no study has comprehensively evaluated the nasal microbiota in HD patients using advanced sequencing technologies.

In this prospective study, we profiled the microbiota in the anterior nares of 31 HD patients and compared the microbiota to 45 peritoneal dialysis (PD) patients and 22 healthy controls (Control) (subjects who presented for potential living kidney donation). Full details of demographical information including age, sex, race, ethnicity, type of dialysis, and hemodialysis access can be found in the Supplementary Material (Table S1). The Weill Cornell institutional review board approved this protocol (IRB#1604017181); all participants provided written informed consent; and our study adheres to the Declaration of Helsinki. Full details of the microbiome profiling can be found in the supplementary methods (Item S1). Briefly, anterior nasal swab specimens were collected from the participants with a Copan Eswab. The DNA was isolated using a Promega Maxwell DNA extraction kit. Polymerase chain reaction was performed to amplify the V4-V5 hypervariable region of the 16S rRNA gene. The amplicon libraries were sequenced using an Illumina MiSeq instrument.

We obtained a total of 3,583,383 amplicon sequence variants with a median of 34,950 amplicon sequence variants and interquartile range of 24,960 and 45,700 amplicon sequence variants per sample. The nasal microbial diversity as measured by Chao1 index, a measure of the estimated number of different amplicon sequence variants, was higher in HD patients than in the Control subjects (median 43 vs 31.5, respectively; *P* = 0.097) but similar in HD patients compared with PD patients (median 43 vs 45.5, respectively, *P* = 0.66). The nasal microbial diversity as measured by the Shannon diversity index was similar among HD patients compared with Control subjects (2.2 vs 1.8, respectively, *P* = 0.31) and HD patients compared with PD patients (2.2 vs 2.1, respectively, *P* = 0.54).

The most common genera in the nasal microbiota cohort (>2% mean relative abundance over all specimens) included: *Staphylococcus, Arthrobacter, Corynebacterium, Cutibacterium, Anaerococcus,* and *Streptococcus.* Among the HD cohort, 68% had at least 25% relative abundance of *Staphylococcus* in their anterior nares. The full profiles of the nasal microbiota are found in Fig 1 along with 4 negative controls. The negative controls all show a significant amount of *Arthrobacter,* suggesting that the presence of this genus is a contaminant. We compared the relative abundance of these top genera between HD patients to Control subjects and HD patients to PD patients. The relative abundance of *Staphylococcus* was significantly higher in HD patients than in Control subjects (median 0.390 vs 0.161, Wilcoxon *P* value 0.01, adjusted *P* value 0.07) but similar in HD patients compared with PD patients (median 0.390 vs 0.275, *P* value 0.60, adjusted *P* value 0.92) (Table 1). The relative abundance of *Streptococcus* was significantly higher in HD patients than in Control subjects (median 0.008 vs 0.002, respectively, *P* value 0.04, adjusted *P* value 0.08) but similar in HD patients and in PD patients (median 0.008 vs 0.011, respectively, *P* value 0.92, adjusted *P* value 0.92) (Table 1). The relative abundances of *Anaerococcus, Corynebacterium,* and *Cutibacterium* were not significantly different between HD patients and Control subjects or between HD patients and PD patients (Table 1). Within the HD group, 5 HD patients had *Staphylococcus* bacteremia at the time of nasal swab and 26 did not; 1 HD patient had *Streptococcus* bacteremia at the time of nasal swab and 30 patients did not. The nasal abundance of *Staphylococcus* was numerically higher in the HD patients who developed *Staphylococcus* bacteremia than in the HD patients who did not (median 0.408 vs 0.289, respectively, *P* = 0.42, Wilcoxon rank sum test).

**Figure 1:**
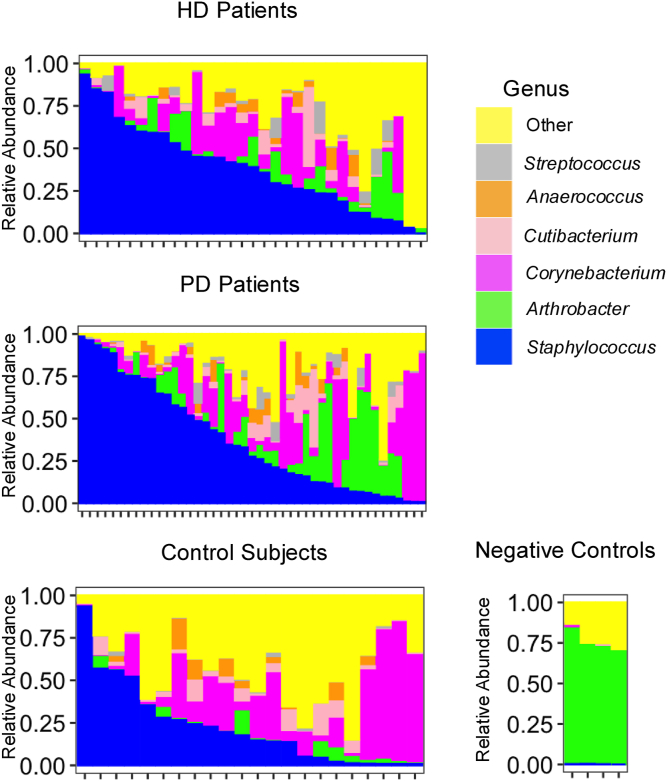
Nasal microbiota profiles across HD patients, PD patients, and Control subjects. Each set of the bar graphs represent individual microbiota profiles in each group (HD patients, left top panel; PD Patients, left middle panel; Control subjects, left bottom panel; and negative controls, right bottom panel). On the x-axis is the individual specimens and on the y-axis is the relative abundance of taxa with the genera represented by different colors as specified in the legend.

**Table 1:** Comparison of the Relative Abundances of the top Genera Between the Hemodialysis (HD) Group, Peritoneal Dialysis (PD) Group, and the Healthy Control (HC) Group

Genus	HD Group Median Abundance n = 31	PD Group Median Abundance n = 45	HC Group Median Abundance n = 22	HD vs HC Wilcoxon *P*	HD vs HC Adjusted *P*	HD vs PD Wilcoxon *P*	HD vs PD Adjusted *P*
*Anaerococcus*	0.016	0.009	0.008	0.75	0.75	0.90	0.92
*Arthrobacter*	0.039	0.083	0.019	0.04	0.08	0.36	0.92
*Corynebacterium*	0.085	0.080	0.165	0.14	0.21	0.63	0.92
*Cutibacterium*	0.013	0.007	0.016	0.19	0.23	0.90	0.92
*Staphylococcus*	0.390	0.275	0.161	0.01	0.07	0.60	0.92
*Streptococcus*	0.008	0.011	0.002	0.04	0.08	0.92	0.92

*Note:* Wilcoxon *P* values were calculated using the Wilcoxon rank sum test. Adjusted *P* values were adjusted using Benjamini-Hochberg correction.

Our study provides a more complete characterization of the nasal microbiota in HD patients. We have previously reported that PD patients have a distinct microbiota signature associated with an increased *Streptococcus* nasal abundance.^5^ In a similar way, HD patients also have an increased *Staphylococcus* and *Streptococcus* nasal abundance when compared to Control subjects but similar *Staphylococcus* and *Streptococcus* nasal abundance compared with PD patients. Whether a predominant nasal microbiota with *Staphylococcus* and *Streptococcus* abundance is associated with infectious complications is not known in HD patients but previous studies on *Staphylococcus* colonization suggests this potential relationship.^1^ Our limited pilot data supports this relationship and indicates a higher nasal abundance of *Staphylococcus* in HD patients who have concurrent *Staphylococcus* bacteremia. Limitations of our study include the cross-sectional nature of the study. Another important limitation is that our 16S gene rRNA sequencing cannot assess species level identification, particularly for *Staphylococcus.* Despite these limitations, our study provides a first-in-kind characterization of the nasal microbiota in HD patients.

## Supplementary Materials

Supplementary File (PDF) Item S1; Table S1.
