# Supplementary material for: A Staphylococcus Dominant Nasal Microbiota in Hemodialysis Patients
Source: Kidney Med. 2025 Sep 19;7(10):101079. doi: 10.1016/j.xkme.2025.101079 (PMC12495457; doi:10.1016/j.xkme.2025.101079)
Supplement: Supplementary File (PDF) — Item S1; Table S1. [file mmc1.pdf]

Item S1. Supplementary Methods

DNA Extraction and Sequencing

An aliquot of a fecal specimen is combined into a Qiagen PowerBead glass 0.1 mm tube (13118-50). Using a Promega Maxwell RSC PureFood GMO and Authentication Kit (AS1600), 1mL of CTAB buffer & 20µl of RNAse A Solution is added to the PowerBead tube. The sample is then vortexed for 10 seconds and incubated at 60°C for 10 minutes on an Eppendorf ThermoMixer F2.0, shaking at 1500 rpm. The tube is attached to a horizontal microtube attachment on a Vortex Genie2 (SI-H524) and vortexed at high-speed for 20 minutes. The sample is then centrifuged on an Eppendorf Centrifuge 5430R at 40°C, 12700 rpm for 10 minutes, and centrifuged again to eliminate foam for an additional 10 minutes. Any foam is removed using a pipette. The tube is added to a Promega MaxPrep Liquid Handler tube rack. The Liquid Handler instrument is loaded with proteinase K tubes, lysis buffer, elution buffer, 1000mL tips, 50mL tips, 96-sample deep-well plate, and Promega Maxwell RSC 48 plunger tips. The Promega MaxPrep Liquid Handler instrument is programed to transfer all sample lysate (300µl) into Promega Maxwell RSC 48 extraction cartridge for DNA extraction. The extraction cartridge is loaded into Promega Maxwell RSC 48 for DNA extraction and the eluted DNA is transferred to a standard 96-well plate. DNA is quantified using Quant-iT dsDNA High Sensitivity Assay Kit using Promega GloMax Discover plate reader on a microplate (655087). Library generation was created using the Earth Microbiome Project. Amplicon libraries are washed using Beckman Coulter AMPure XP magnetic beads. Library quality & size verification is analyzed using PerkinElmer LabChip GXII instrument with DNA 1K Reagent Kit (CLS760673). Library concentrations are further

quantified using Quant-iT dsDNA High Sensitivity Assay Kit using Promega GloMax Discover plate reader on a microplate (655087). Library molarity is calculated based on library peak size & concentration and are normalized to 2nM using the Hamilton Microlab NIMBUS and pooled together using the same volume across all normalized libraries into a 1.5ml Eppendorf DNA tube (022431021). Pooled libraries are sequenced on the Illumina MiSeq instrument at loading concentration of 3pM with 15% PhiX, paired-end 250 using MiSeq Reagent Kit v2, 500-cycles (MS-102-2003).

Bioinformatics

Demultiplexed raw reads were processed using the Nextflow (Di Tommaso et at., 2017) nf-core (Ewels et al, 2020) ampliseq pipeline (Straub et al., 2020), version 2.6.1, with the following parameters: -profile singularity --input SampleSheet.tsv --FW_primer GTGYCAGCMGCCGCGGTAA --RV_primer CCGYCAATTYMTTTRAGTTT --metadata Metadata.tsv --outdir results --dada_ref_taxonomy silva --ignore_empty_input_files --ignore_failed_trimming --min_frequency 10 --retain_untrimmed --trunclenf 240 --trunclenr 160. Specifically, reads were trimmed with cutadapt (Martin, 2011), PhiX and quality filtering, read pair merging, and amplicon sequence variant resolution was analyzed with DADA2 (Callahan et al., 2016). Subsequent taxonomic assignment was also analyzed with DADA2, using the Silva reference database (Quast et al., 2012), version 138.

Supplementary References

Callahan, B. J., McMurdie, P. J., Rosen, M. J., Han, A. W., Johnson, A. J. A., & Holmes, S. P. (2016). DADA2: High-resolution sample inference from Illumina amplicon data. Nature methods, 13(7), 581-583.

Di Tommaso, P., Chatzou, M., Floden, E. W., Barja, P. P., Palumbo, E., & Notredame, C. (2017). Nextflow enables reproducible computational workflows. Nature biotechnology, 35(4), 316-319.

Ewels, P. A., Peltzer, A., Fillinger, S., Patel, H., Alneberg, J., Wilm, A., ... & Nahnsen, S. (2020). The nf-core framework for community-curated bioinformatics pipelines. Nature biotechnology, 38(3), 276-278.

Martin, M. (2011). Cutadapt removes adapter sequences from high-throughput sequencing reads. EMBnet. journal, 17(1), 10-12.

Quast, C., Pruesse, E., Yilmaz, P., Gerken, J., Schweer, T., Yarza, P., ... & Glöckner, F. O. (2012). The SILVA ribosomal RNA gene database project: improved data processing and web-based tools. Nucleic acids research, 41(D1), D590-D596.

Straub, D., Blackwell, N., Langarica-Fuentes, A., Peltzer, A., Nahnsen, S., & Kleindienst, S. (2020). Interpretations of environmental microbial community studies are biased by the selected 16S rRNA (gene) amplicon sequencing pipeline. Frontiers in microbiology, 2652.

Supplementary Results

Table S1: Demographics and Outcomes Table

Characteristic	PD Cohort (n=45) median or proportion	HD Cohort (n = 31) median or proportion	HC Cohort (n = 22) median or proportion
Age, years	61	64	51
Female Sex	24 (53%)	11 (35%)	13 (59%)
Ethnicity			
Hispanic	7 (16%)	7 (23%)	3 (14%)
Non-Hispanic	36 (80%)	24 (77%)	16 (73%)
Declined	2 (4%)	0 (0%)	3 (14%)
Race			
Asian	6 (13%)	6 (19%)	1 (5%)
Black	19 (42%)	10 (32%)	5 (23%)
White	12 (27%)	8 (26%)	11 (50%)
Other	6 (13%)	7 (23%)	1 (5%)
Declined	2 (4%)	0 (0%)	4 (18%)
History of Hypertension	39 (87%)	31 (100%)	2 (9%)
History of Diabetes Mellitus	16 (36%)	18 (58%)	0 (0%)
Automated Peritoneal Dialysis	30 (67%)		
HD Access - Dialysis Catheter		13 (42%)	
Concurrent *Staphylococcus* bacteremia		5 (16%)	
Concurrent *Streptococcus* bacteremia		1 (3%)	
Mortality in 1 year	2 (4%)	1 (3%)	
